# Refixierung von osteochondralen Flake-Frakturen nach Patellaluxation – die Parachute-Technik

**DOI:** 10.1007/s00064-024-00873-7

**Published:** 2024-11-06

**Authors:** Romed P. Vieider, Sebastian Siebenlist, Lorenz Fritsch, Ahmed Ellafi, Yannick Ehmann, Julian Mehl

**Affiliations:** Sektion Sportorthopädie, Klinikum rechts der Isar, Technische Universität München, Ismaninger Str. 22, 81675 München, Deutschland

**Keywords:** Knie, Patella, Knorpelschaden, Trauma, Knorpeltherapie, Knee, Patella, Cartilage defect, Trauma, Cartilage repair

## Abstract

**Operationsziel:**

Patellaluxationen sind eine häufige Entität in der orthopädischen Praxis und gehen in bis zu 58 % der Fälle mit osteochondralen Frakturen der retropatellaren Knorpeloberfläche, sog. Flake-Frakturen, einher. Die Parachute-Technik stellt eine einfache und kosteneffektive chirurgische Option dar, die im Rahmen der operativen Versorgung dieser Verletzung darauf abzielt, die osteochondrale Integration wiederherzustellen und den nativen Knorpel zu erhalten.

**Indikationen:**

Flake-Fraktur der Patella mit osteochondralen Fragmenten.

**Kontraindikationen:**

Patellafraktur.

**Operationstechnik:**

Durch den Einsatz von transpatellaren, resorbierbaren Nahtmaterialien wird eine stabile osteochondrale Schnittstelle erreicht, ohne das Fragment selbst zu durchdringen.

**Weiterbehandlung:**

Die postoperative Behandlung beinhaltet eine teilweise Belastung mit maximal 20 kg für 6 Wochen in vollständiger Kniestreckung. Zusätzlich ist der Bewegungsumfang der Kniebeugung auf 30° begrenzt und wird alle 2 Wochen um 30° erhöht.

**Ergebnisse:**

Zur Beurteilung der kurz- bis mittelfristigen klinischen Ergebnisse wurden alle Patienten mit akuter Patellaluxation eingeschlossen, die zwischen 01/2012 und 11/2022 mittels Parachute-Technik versorgt wurden. Die klinischen Ergebnisse wurden mit der visuellen Analogskala (VAS), der Tegner-Aktivitätsskala (TAS), dem Kujala-Score, dem Knee Injury and Osteoarthritis Outcome Score (KOOS) und dem International Knee Documentation Committee (IKDC) erhoben. Von insgesamt 20 Patienten konnten 19 (10 männlich, 11 rechtsseitig, 95 % Follow-up-Rate) für die Nachuntersuchung erreicht werden. Der durchschnittliche Follow-up-Zeitraum betrug 62,5 ± 20,5 Monate. Die klinischen Outcome-Scores resultierten in folgenden Ergebnissen: VAS 0,5 ± 1,6; TAS 5,8 ± 2,2; Kujala 89,4 ± 12,5; KOOS 87,8 ± 14,1 und IKDC 86,7 ± 14,3. Insgesamt gaben 18 Patienten (90,0 %) an, dass sie den Eingriff erneut durchführen lassen würden. Zum Zeitpunkt der Nachuntersuchung waren 19 Patienten (95,0 %) mit dem Operationsergebnis zufrieden. Ein Patient (männlich, 23 Jahre alt) benötigte eine Revision. Keiner der eingeschlossenen Patienten hatte eine erneute Patellaluxation. Zusammenfassend lässt sich schließen, dass die Parachute-Technik bei akuter Patellaluxation eine sehr gute klinische Funktion im kurz- bis mittelfristigen Follow-up aufweist.

## Abstract (en)

**Objective:**

Patellar dislocations are a common occurrence in orthopedic practice, often accompanied by osteochondral fractures of the retropatellar cartilage surface, known as flake fractures, in up to 58% of cases. The parachute technique represents a simple and cost-effective surgical option aimed at restoring osteochondral integration and preserving native cartilage.

**Indications:**

Flake fracture of the patella with osteochondral fragments.

**Contraindications:**

Patella fracture.

**Surgical technique:**

By utilizing transpatellar, absorbable sutures, a stable osteochondral interface is achieved without penetrating the fragment itself.

**Postoperative management:**

Postoperative treatment involves partial weight-bearing with a maximum of 20 kg for 6 weeks in full knee extension. In addition, the range of motion of knee flexion is limited to 30° and is increased by 30° every 2 weeks.

**Results:**

To examine the short- to medium-term clinical outcomes, all patients with acute patellar dislocation treated using the parachute technique between 01/2012 and 11/2022 were included. Clinical outcomes were assessed using the visual analog scale (VAS), Tegner Activity Scale (TAS), Kujala Score, Knee Injury and Osteoarthritis Outcome Score (KOOS), and International Knee Documentation Committee (IKDC). Out of 20 patients, 19 (10 men, 11 right-sided, 95% follow-up rate) could be recruited for postoperative evaluation. The average follow-up period was 62.5 ± 20.5 months. The clinical outcome scores yielded the following results: VAS 0.5 ± 1.6, TAS 5.8 ± 2.2, Kujala 89.4 ± 12.5, KOOS 87.8 ± 14.1, and IKDC 86.7 ± 14.3. Overall, 18 patients (90.0%) expressed willingness to undergo the procedure again. At the time of follow-up, 19 patients (95.0%) were satisfied with the surgical outcome. One patient (23-year-old man) required revision. None of the included patients suffered from the recurrence of patellar dislocation. In summary, the parachute technique demonstrated excellent clinical function in the short- to medium-term follow-up for acute patellar dislocation.

## Vorbemerkungen

Patellaluxationen stellen im orthopädischen Alltag eine häufige Entität dar, und die veröffentlichten Inzidenzzahlen reichen von 13,5 bis 53,6 pro 100.000 Personenjahre. Die primäre Luxation tritt meist im Jugendalter auf [10, 11].

Begleitende Knorpelverletzungen werden in bis zu 95 % der Fälle berichtet. Osteochondrale Frakturen der Kniescheibe, sog. Flake-Frakturen, treten in bis zu 58 % der Fälle auf [7, 11] Es gilt der Konsens, dass diese osteochondralen Defekte behandelt werden, um das Risiko einer patellofemoralen Arthrose zu vermindern. Abhängig von Größe und Lokalisation des Defekts sind hierfür verschiedene Verfahren beschrieben [1, 6]. Zusätzlich sollte in Zusammenschau der patientenindividuellen Risikofaktoren die Stabilisation der Patella diskutiert werden [3].

Die Parachute-Technik ist eine simple Operationsmethode, um den nativen Knorpel zu erhalten. Mithilfe von transpatellaren, resorbierbaren Fäden wird eine stabile Knorpel-Knochen-Situation im Defektbett erzielt, ohne das Fragment zu durchbohren [8].

## Operationsprinzip und -ziel

Die Parachute-Technik stellt ein einzeitiges Operationsverfahren zur Refixierung von osteochondralen, patellaren Flake-Frakturen nach Patellaluxation dar. Unter Verwendung von resorbierbaren Fäden können osteochondrale Fragmente wieder stabil und mit gleichmäßigem Anpressdruck im retropatellaren Defektbett refixiert werden. An dieser beschriebenen Technik ist einzigartig, dass keine Penetration des Knorpel-Knochen-Fragments erforderlich ist.

## Vorteile

- Die Parachute-Technik ermöglicht die stabile Refixierung des nativen osteochondralen Fragments in sein Defektbett.
- Durch die frei wählbare Konfiguration der Bohrlöcher kann die Technik unabhängig von der Form des Fragments angewendet werden.
- Im Vergleich zu punktuell fixierenden Verfahren (Schrauben, Stifte) ermöglicht die Fadenkonfiguration einen flächigen Anpressdruck des Fragments in das Defektbett, um eine optimale Heilung zu ermöglichen.
- Durch die flächige Kompression kann die Indikation auch auf Flakes mit keiner oder sehr geringen Knochenschuppen ausgeweitet werden.
- Die Durchführung der Technik benötigt kein spezielles Instrumentarium oder Material, sie lässt sich mit wenigen Standardwerkzeugen durchführen.
- Regenerative Knorpelverfahren stellen bei erfolgloser Refixierung weiterhin eine Option dar.

## Nachteile

- Die osteochondrale Refixierung mit Fäden in Parachute-Konfiguration kann nicht für jedes osteochondrale Fragment angewendet werden.
- Bei einer gleichzeitigen Rekonstruktion des medialen patellofemoralen Haltebandes mit patellaren Knochentunneln besteht die potenzielle Gefahr des Tunnelkonflikts.
- Die Frühphase im postoperativen Verlauf kann von retropatellaren Krepitationen durch das Fadenmaterial begleitet sein.

## Indikationen

**Abb. 1:**
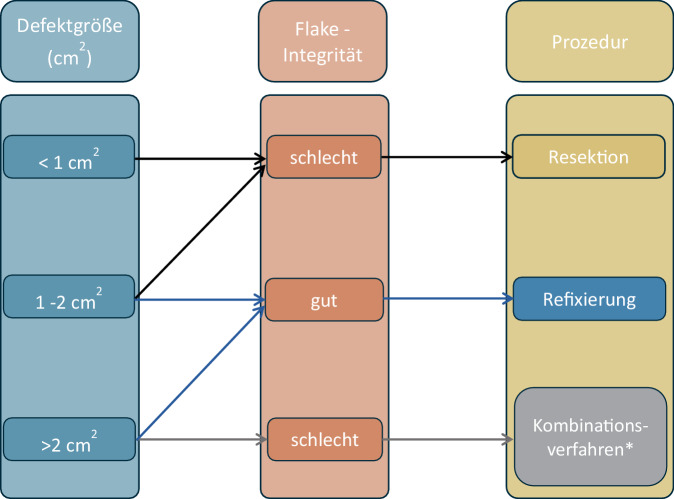
Behandlungsalgorithmus zur Therapieentscheidung bei osteochondralen Flake-Frakturen, abhängig von der Größe des Defekts und der Integrität des Flakes. *Asterisk* Das Kombinationsverfahren besteht zum einen aus der Resektion des Knorpel-Flakes und einem zusätzlichen knorpelregenerativen Verfahren

- Die Indikation basiert auf einer Kombination aus Anamnese, klinischer Untersuchung und der radiologischen Bildgebung. Bei der Flake-Fraktur können osteochondrale Fragmente v. a. auf magnetresonanztomographischen (MRT) Aufnahmen vorab auf Größe und Integrität beurteilt werden.
- Die Autoren empfehlen, vor dem offenen Eingriff eine diagnostische Arthroskopie durchzuführen, um das Fragment zu bergen, das Defektbett zu beurteilen und anhand dieser Befunde die Indikation zur Refixierung zu bestätigen (Abb. 1).

## Kontraindikationen

- Patellafraktur

## Patientenaufklärung

- Allgemeine Operationsrisiken
- Bruch des Fragments während der Refixierung
- Verlust des Fragments beim Auswaschen/Spülen während der Arthroskopie (aufgrund der Größe des Flakes sehr unwahrscheinlich)
- Die Gefahr der Patellafraktur kann durch mehrere, transpatellare Tunnel erhöht werden
- Durch das Fadenmaterial können retropatellare Krepitationen auftreten
- Fremdkörpergefühl an der Verknotungsstelle ventralseitig der Patella

## Operationsvorbereitungen

- Röntgenaufnahmen a.-p. und lateral, um Frakturen auszuschließen
- Ganzbeinaufnahmen zur Bemessung der Beinachse
- MRT zur Evaluierung von Begleitverletzung und ersten Einschätzung des osteochondralen Fragments
- Klinische Untersuchung

## Instrumentarium

- Standardoperationsinstrumente
- Resorbierbares Fadenmaterial, in dieser Technikbeschreibung wurden resorbierbare Vicryl-Fäden (Vicryl USP 1/0, Ethicon Inc., Bridgewater, NJ, USA) verwendet (Tab. 1)
- Flexibler Führungsdraht, um die Fäden transpatellar zu shuttlen (Nitinol Suture Passing Wire, Arthrex, Naples, FL, USA)
- Arthroskopie (nicht obligat)

**Tab. 1:** Operationsspezifische Instrumente und Materialien

Instrumente und Material	Empfohlene Verwendung
Kirschner-Drähte	1,2 mm zur temporären Flake-Stabilisation 1,4 mm für die transpatellaren Tunnel 2,0 mm Draht als „Joystick“ für die laterale Eversion der Patella und den erleichterten Zugang zum Defekt
Flexibler Faden-Führungsdraht (Nitinol Suture Passing Wire, Fa. Arthrex, Naples, FL, USA)	Transpatellares Shuttlen der Fäden
Resorbierbares Fadenmaterial (Vicryl USP 1/0, Ethicon Inc., Bridgewater, NJ, USA)	Transossäre Flake-Refixierung

## Anästhesie und Lagerung

- Die Operation kann in Allgemein- oder Regionalanästhesie durchgeführt werden.
- Die Autoren empfehlen die Durchführung des Eingriffs in Rückenlagerung mit einer Beinstütze und einer Fußrolle, um das Bein in 90°-Knieflexion aufstellen zu können.
- Zusätzlich wird empfohlen, die Option zu wahren, das Bein auch in 30°-Knieflexion aufstellen zu können. Dies kann durch eine verstellbare Fußrolle oder beispielsweise eine aufgestellte Tuchrolle als Unterstützung erreicht werden.

## Operationstechnik

**Abb. 2:**
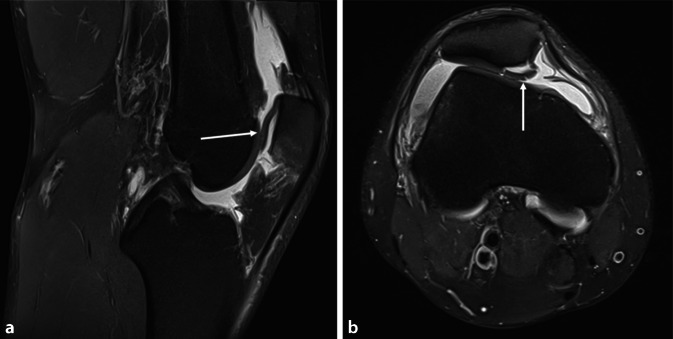
Die präoperative Magnetresonanztomographie zeigt ein rechtes Kniegelenk eines 25-jährigen Patienten nach traumatischer Patellaerstluxation. Der Knorpel zeigt sich über nahezu zwei Drittel der sagittalen Ausdehnung abgehoben (**a**). Typischerweise tritt der Schaden (*weißer Pfeil*) an der medialen Patellafacette auf (**b**)

**Abb. 3:**
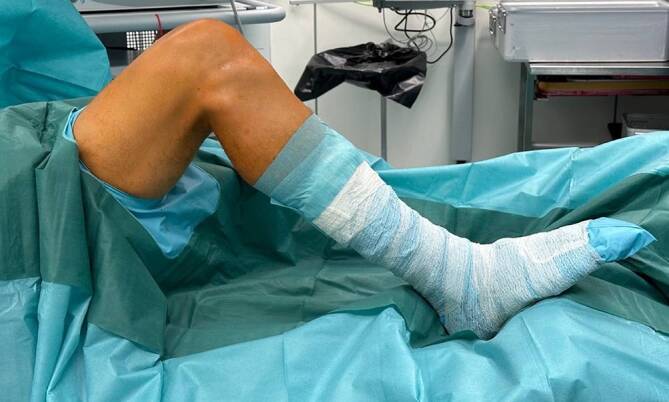
Der Patient wird auf dem Rücken gelagert. Die seitliche Beinstütze sowie die Fersenrolle ermöglichen das Aufstellen des Beins in 90° für die diagnostische Arthroskopie. Die Autoren empfehlen eine Vorrichtung, die es erlaubt, das Bein während der Operation auch auf 30°-Knieflexion einzustellen. Dies kann auch mittels steriler Tuchrolle erfolgen

**Abb. 4:**
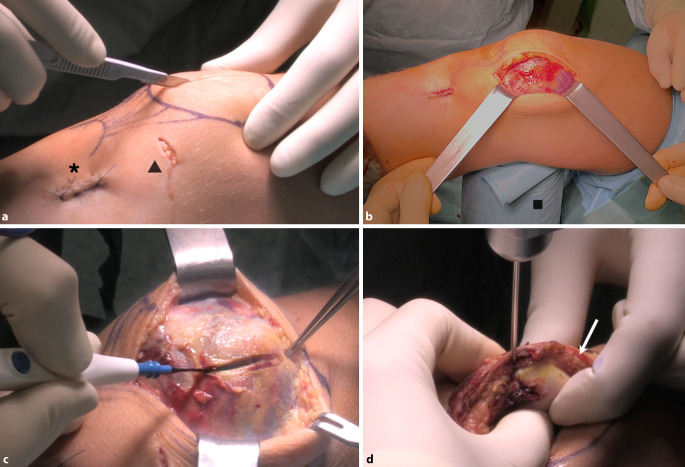
Blick von lateral auf ein rechtes Kniegelenk. Nach dem Anzeichnen der anatomischen Landmarken (Patella, Tuberositas tibiae und Patellasehne) wird ein zentraler, paramedianer Hautschnitt angelegt (**a**). Es erfolgt die Präparation bis auf die Gelenkkapsel bzw. deren Übergang zum M. vastus medialis (**b**). Die sterile Tuchrolle dient als Vorrichtung, um das Bein in 30° Beugung zu halten (*Viereck*). Dadurch lassen sich die Gewebeschichten bei der Präparation einfacher trennen. Um den Zugang zur retropatellaren Knorpelfläche zu ermöglichen, wird die Gelenkkapsel mittels Elektrokauter durchtrennt (**c**). Diese soll nicht zu knapp vom medialen Rand der Patella präpariert werden (nicht < 5 mm), um mit dem Gewebe den Kapselverschluss nach dem Eingriff zu erleichtern (*Pfeil* in **d**). Anschließend wird unter Darstellung des medialen Patellarandes ein Kirschner-Draht (Stärke 2 mm) zentral von medial in die Patella eingebracht (**d**). Dieser ermöglicht eine schonende und stabile Eversion der Patella während der gesamten Prozedur. Durch die 30°-Beugung im Kniegelenk wird zudem eine geringe „Gegenspannung“ erzeugt, welche die Patella beim Bohren zusätzlich stabilisiert. *Asterisk* Entnahmestelle der Sehne des M. gracilis, *Dreieck* mediales Arbeitsportal der vorangegangenen Arthroskopie

**Abb. 5:**
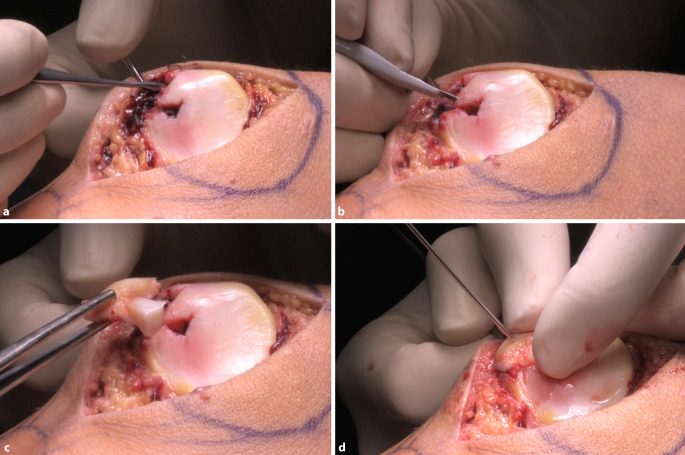
Das Defektbett wird im nächsten Schritt vorsichtig mit beispielsweise einem scharfen Löffel vom Hämatom befreit (**a**), und instabile Ränder werden ggf. scharf abgetragen (**b**). Durch das intraartikuläre Milieu verursachen osmotische Prozesse ein Aufquellen des freien Fragments. In diesem Fall empfiehlt sich die Trimmung des Fragments auf die Defektbettgröße mit einem scharfen Skalpell. Das Fragment (**c**) wird mit einem Kirschner-Draht der Stärke 1,2 mm nun durchbohrt und passgenau in das Defektbett eingesetzt. Zur temporären Fixierung wird der Draht weiter in die Patella vorgebohrt (**d**)

**Abb. 6:**
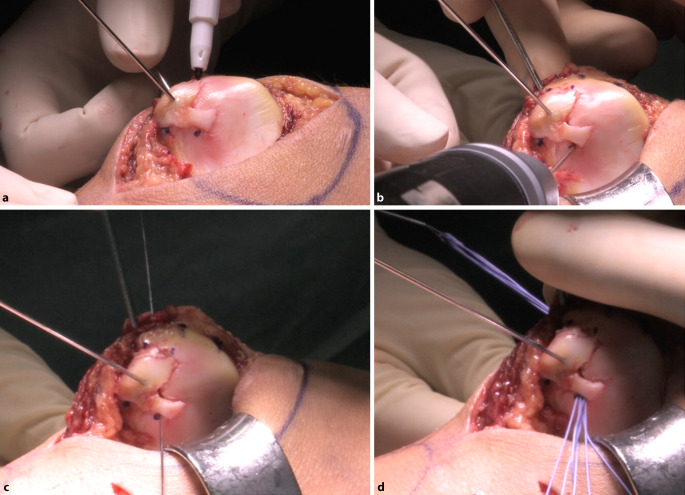
Die Anlage der vorab mit dem Stift markierten Eintrittspunkte (**a**) der transpatellaren Tunnel erfolgt mittels 1,4 mm starken Kirschner-Drahts. Die Tunnel werden exakt an den Grenzen des osteochondralen Fragments zum gesunden Knorpel angelegt (**b**). Die Austrittspunkte an der ventralen Seite der Patella sollten in konfluierender Konfiguration erfolgen. Die Vicryl-Fäden werden mit dem flexiblem Faden-Führungsdraht (Nitinol Suture Passing Wire, Fa. Arthrex, Naples, Florida, USA) durch das angelegte Bohrloch geführt (**c**). Der Erfahrung der Autoren zufolge können bis zu 6 Fäden pro Tunnel geführt werden (**d**). Die Druckverteilung pro Faden wird somit deutlich reduziert, und ein Einschneiden des Fadens durch den Fragmentknorpel kann verhindert werden

(Abb. 2, 3, 4, 5, 6, 7 und 8)

**Abb. 7:**
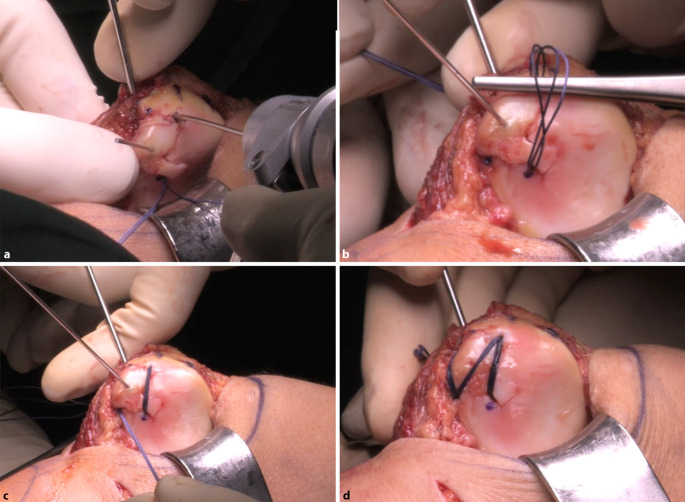
Die Positionierung der Tunnel richtet sich nach der Konfiguration des Defektbetts (**a**). Nach Anlage der weiteren Tunnel können die Fäden gleichmäßig eingezogen werden. Hierbei empfiehlt sich die Verwendung einer Pinzette als Hypomochlion (**b**). Die Bohrungen sowie das Einziehen der Fäden werden so oft wiederholt, bis die gewünschte Konfiguration der Fäden erreicht wurde (**c**). Diese sollten so angelegt werden, dass ein gleichmäßiger Anpressdruck des Fragments in das Defektbett erzielt wird (**d**)

**Abb. 8:**
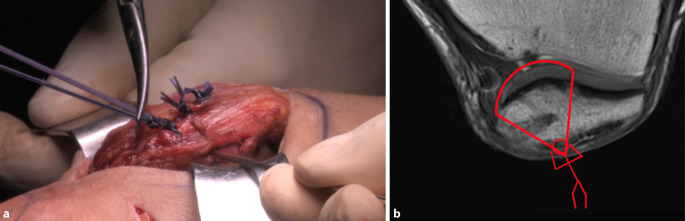
An der ventralen Seite der Patella werden die Fäden verknotet (**a**). Die Endkonfiguration der Fäden (*rot*, **b**) in den konfluierenden Knochentunneln erinnert an einen Fallschirm (engl. „parachute“)

## Postoperative Behandlung

Die postoperative Behandlung beinhaltet eine teilweise Belastung mit maximal 20 kg für 6 Wochen in vollständiger Kniestreckung (Ext). Die Belastung in Kniebeugung ist in dieser Zeit nicht erlaubt. Zusätzlich ist der Bewegungsumfang (engl.: „range of motion“ [ROM]) der Kniebeugung (Flex) auf 30° begrenzt und wird alle 2 Wochen nach dem folgenden Schema um 30° erhöht:

- Wochen 1 und 2: ROM Ext/Flex 0°/0°/30°,
- Wochen 3 und 4: ROM Ext/Flex 0°/0°/60°,
- Wochen 5 und 6: ROM Ext/Flex 0°/0°/90°.

Anschließend soll die vorsichtige Steigerung der Belastung bis zum vollständigen Körpergewicht und vollen Bewegungsumfang erfolgen.

## Fehler, Gefahren, Komplikationen

- Konfluierende Knochentunnel bzw. Tunnelkonflikt bei gleichzeitiger medialer patellofemoraler Ligament(MPFL)-Rekonstruktion: Dies könnte zum einen zu einer Lockerung der Anker der MPFL-Rekonstruktion oder zu einer Durchtrennung der transpatellaren Fäden bei der Ankeranlage für die patellare Befestigung der MPFL-Rekonstruktion am medialen Rand der Patella führen.
  - Strategie zur Vermeidung: Bohrkanäle für die MPFL-Rekonstruktion werden vor den transpatellaren Bohrungen durchgeführt, so kann im Zweifelsfall überprüft werden, ob ein Tunnelkonflikt vorliegt.
- Patellafraktur durch mehrere Knochentunnel in einer Ebene oder durch vorgeschädigte Patella aufgrund des Luxationstraumas
- Knorpelausbruch bei fehlgeschlagener Einheilung im postoperativen Verlauf

## Ergebnisse

Insgesamt wurden 20 konsekutive Patienten mit akuter Patellaluxation (10 männlich, 11 rechts), die zwischen 01/2012 und 11/2022 mit der beschriebenen Operationstechnik und zusätzlich einer Rekonstruktion des medialen patellofemoralen Ligaments versorgt wurden, eingeschlossen. Davon konnten 19 Patienten (95 % Follow-up-Rate) mit einem mittleren Follow-up von 62,5 ± 20,5 Monaten für die Nachuntersuchung erreicht werden. Die detaillierten demografischen Daten können aus Tab. 2 entnommen werden.

**Tab. 2:** Demografie und Follow-up-Zeitraum der in die Nachuntersuchung eingeschlossenen Patienten, die bei akuter Flake-Fraktur nach Patellaluxation mittels osteochondraler Flake-Refixierung mit der beschriebenen Parachute-Technik versorgt wurden

Parameter	Wert	SD	Spannweite
Alter bei OP (a)	20,8	±5,3	14,0–30,6
BMI (kg/m^2^)	24,7	±6,7	18,1–51,3
Follow-up (Mo)	62,2	±20,5	27,0–111,3
Geschlecht (%)	10 m	10 w	–
Seite	9 li	11 re	–

*OP* Operation, *m* männlich, *w* weiblich, *re* rechts, *li* links

Die funktionellen Ergebnisse wurden mit den folgenden klinischen Outcome-Scores erhoben: visuelle Analogskala (VAS), Tegner Aktivitätsskala (TAS) [4], Kujala Score [2], Knee Injury and Osteoarthritis Outcome Score (KOOS) [9], International Knee Documentation Committee (IKDC, Tab. 3; [5]).

**Tab. 3:** Klinische Outcome-Scores: Visual Analogue Scale (VAS), Tegner Activity Scale (TAS), Knee Injury and Osteoarthritis Outcome Score (KOOS), International Knee Documentation Committee (IKDC)

**Parameter**	**Wert**	**SD**	**Spannweite**
**VAS **_**(Follow up)**_	0,5	±1,6	0–7
**TAS**	5,8	±2,2	2–9
**Kujala-Score**	89,4	±12,5	60–100
**KOOS**_**Gesamt**_	87,8	±14,1	52–100
KOOS_Symptome_	84,6	±13,7	60–100
KOOS_Schmerz_	92,8	±11,5	58–100
KOOS_ADL_	94,6	±13,8	43–100
KOOS_Sport_	84,7	±21,0	35–100
KOOS_QOL_	81,9	±24,7	25–100
**IKDC**	86,7	±14,3	54–100
**Würden Sie die Operation nochmals durchführen lassen?**			
*Ja*	18 (90,0 %)	*Nein*	2 (10,0 %)
**Waren Sie mit dem Ergebnis der Operation zufrieden?**			
*Ja*	22 (100 %)	*Nein*	0 (0 %)
**Trat seit der Operation eine erneute Patellaluxation auf?**			
*Ja*	0 (0 %)	*Nein*	22 (100 %)

Insgesamt gaben 18 Patienten (90,0 %) an, dass sie sich der Operation erneut unterziehen würden. Zum Zeitpunkt der Nachuntersuchung waren 19 (95,0 %) mit dem Operationsergebnis zufrieden. Ein Patient (männlich, 23 Jahre) musste 3 Monate nach der initialen Operation revidiert und mit einem knorpelregenerativen Verfahren („minced cartilage“) versorgt werden. Bei keinem der eingeschlossenen Patienten trat eine weitere Patellaluxation auf.

Zusammenfassend zeigten sich klinisch und funktionell zufriedenstellende Ergebnisse in der untersuchten Kohorte. Die Parachute-Technik bietet sich als einzeitige, unkomplizierte und kostengünstige Option zur Refixierung osteochondraler Flake-Frakturen nach traumatischer Patellaluxation an.
